# Development and Validation of a Brief Warzone Stressor Exposure Index

**DOI:** 10.1177/10731911241298083

**Published:** 2024-12-05

**Authors:** Frederick Anyan, Andreas Espetvedt Nordstrand, Odin Hjemdal, Line Rønning, Ann Hergatt Huffman, Laura K. Noll, Christer Lunde Gjerstad, Robert E. Wickham, Hans Jakob Bøe

**Affiliations:** 1 Norwegian University of Science and Technology, Trondheim, Norway; 2 Norwegian Armed Forces Joint Medical Services, Oslo, Norway; 3 Northern Arizona University, Flagstaff, USA; 4 University of Oslo, Norway

**Keywords:** military personnel, warzone stressors, moral injury, combat trauma, war zone

## Abstract

Existing scales mainly focus on danger-based threats of death and bodily harm to assess exposure to traumatic events in war zone. However, major provocations and transgression of deeply held values and moral beliefs, as well as witnessing the suffering of others can be as traumatic as fear-inducing danger-based events. This raises the need for scales that assess both danger- and nondanger-based events among soldiers operating in modern war zones. Norwegian military personnel deployed to Afghanistan between late 2001 and end of 2020 were invited to participate in a cross-sectional survey with a final sample size of 6,205 (males: *n* = 5,693; 91.7%; mean age = 41.93 years). We applied data reduction techniques (e.g., exploratory factor analysis, EFA, and exploratory graph analysis, EGA, through a community detection algorithm) to develop a 12-item, three-factor model (personal threat, traumatic witnessing, and moral injury) of the Warzone Stressor Exposure Index (WarZEI). Confirmatory factor analysis showed support for the factor model, with evidence of concurrent, discriminant, and incremental validity. These results indicate the WarZEI is a reliable and valid measure for assessing exposure to warzone stressors that allows for heterogeneity and the multidimensional nature of exposure to warzone stressors.

According to the *DSM*-5 (*Diagnostic and Statistical Manual of Mental Disorders* [5th ed.; *DSM-5*; American Psychiatric Association, 2013], traumatic stressors can take the form of actual or threatened physical harm or injury, severe illness, or sexual violence experienced either personally, vicariously as a witness, or remotely and indirectly after the fact. Exposure to such stressors is common, with a lifetime prevalence rate of more than 70% in the civilian population (Kessler et al., 2017). Not surprisingly, military personnel report higher rates of exposure to traumatic stressors (Reger et al., 2019), but also more likely to be exposed to diverse types of stressors (Brownlow et al., 2018). This reflects the broad range of traumatic stressors commonly encountered while serving in a war zone, such as being in life-threatening situations, witnessing moral transgressions, and/or the death and suffering of others, perpetrating violence on others, and potentially losing close comrades in combat action.

Several measures have been used by previous researchers to assess traumatic exposure among military personnel participating in war, but there is no gold standard scale in military traumatic research for assessing exposure to war zone stressors (Porter et al., 2018). Commonly used measures diverge in the number and type of warzone stressors they assess; however, the most extensively used scales focus primarily on threats of death and bodily harm while engaged in combat actions (e.g., Combat Exposure Index; Janes et al., 1991). Other scales such as the Deployment Risk and Resilience Inventory (DRRI; King et al., 2003) were developed as a comprehensive research tool to assess risk and resilience factors pertinent to military deployment stress and its enduring ramifications on veterans’ health. The DDRI contains 201 items covering 14 risk and resilience factors: (a) predeployment/prewar (i.e., prior stressors and childhood family environment), (b) deployment/war zone (i.e., sense of preparedness, difficult living and working environment, concerns about life and family disruptions, deployment social support, sexual harassment, general harassment, perceived threat, combat experiences, exposure to the aftermath of battle, and self-reports of nuclear, biological, or chemical exposures), and postdeployment/postwar (i.e., postdeployment social support and postdeployment stressors) domains. Subsequent improvements led to the development of the 17-item DRRI-2 (Vogt et al., 2013), aiming to ensure its relevance across varied deployment scenarios and bolster its psychometric properties. Notable improvements included the refinement of warfare experience assessment and expanded coverage of familial factors across deployment phases. A recently condensed iteration based on the *combat experiences* and *aftermath of battle* subscales in the broader DRRI-2 resulted in the 9-item Deployment Risk and Resilience Inventory-2 Warfare Exposure—Short Form (DRRI-2 WE-SF; Vogt et al., 2022) introduced to assess direct warfare exposures and their sequelae.

The 7-item Critical Warzone Experiences (CWE; Kimbrel et al., 2014) scale and the 9-item Moral Injury Events Scale (MIES; Nash et al., 2013) are other brief scales relevant for assessment in the veteran populations. The CWE scale was designed to target specific warzone encounters associated with posttraumatic stress disorder (PTSD), anxiety, and depression. The authors noted that the CWE’s direct focus on PTSD, anxiety, and depression is a limitation since many other outcomes such as alcohol misuse might be observed in returning veterans. The two-factor (i.e., perceived transgressions and perceived betrayal subscales) MIES was designed to assess exposure to military events precipitating moral injury (MI) but has faced criticism for its lack of item specificity, potentially undermining its interpretability and yielding inconsistent findings of its measurement model. A three-factor solution (i.e., transgressions-others, transgressions-self, and betrayal) has also been reported by Bryan et al. (2016). In a recent study, Richardson et al. (2020) tested the conceptual replicability of both the two- and three-factor solutions and found support for the two-factor solution.

The diverse array of existing scales for assessing deployment-related experiences exhibits distinct strengths and limitations. Prior to the DRRI-2 WE-SF, the broad and encompassing nature of the DDRI-1 and DDRI-2 posed challenges in large-scale survey administration, potentially elevating respondent burden and data processing costs. Concerns have been raised by researchers regarding the susceptibility to the inclusion of ambiguous items in longer scales, necessitating a shift toward shorter scales to mitigate redundancy and enhance clarity (Allen et al., 2022; Vogt et al., 2013). However, even among existing, empirically validated shorter scales, there are some notable limitations. The DRRI-2 WE-SF, while offering brevity, does not address MI as a distinct domain, unlike the MIES. And while the MIES captures pertinent aspects of MI and traumatic witnessing, it does not capture personal threat experiences. These nuanced distinctions underscore the need for careful consideration of scale selection based on the specific research or intended scope of assessment and the aspects of deployment-related experiences being targeted.

Traditionally, the focus of traumatic stress research has been on fear-based events consisting of danger and horror (Norris, 1990, 1992), in the form of violent encounters with nature, technology, or humankind (Norris, 1992). Such stressors are commonly referred to as personal life threats (Shea et al., 2017; Xue et al., 2015) and are usually described as threatening events precipitating fear-based distress best understood through a neuro-biological model of fear dysregulation (Norrholm & Jovanovic, 2010). In many traumatic situations, however, peritraumatic fear may not be present, and the threat to life or body may not be the most stressful part of the incident (Shakespeare-Finch & Armstrong, 2010; Yehuda et al., 1992). Sensory impressions of death or major suffering of others by seeing, hearing, touching, or smelling can in itself constitute traumatic stressors and are commonly referred to as witnessing stressors (Carson et al., 2000; Dryden, 2012; Fontana et al., 1992; Pietrzak et al., 2011; Stein et al., 2012). Similarly, the unexpected loss of a close other, particularly under traumatic circumstances, has been shown to increase the risk of complicated and prolonged grief reactions (Lundorff et al., 2017). Having had such experiences are more common among military veterans than civilians (Benjet et al., 2016; Wisco et al., 2014), and although the probability of developing PTSD following these events is lower relative to other traumas (Kessler et al., 2017; Wisco et al., 2014), these experiences have been linked with increased debilitating psychological distress, often in the form of guilt and depressive symptoms (Stein et al., 2012).

Some events involving interpersonal harm can also be traumatizing, without a life threat or danger. Instead, the most stressful aspect of some traumatizing experiences may involve major provocations of the individual’s values and morality (Litz et al., 2009). Such moral stressors have been defined as “perpetrating, failing to prevent, bearing witness to, or learning about acts that transgress deeply held moral beliefs and expectations” (Litz et al., 2009, p. 700). The symptom cluster labeled as MI typically does not conform with conceptualizations of posttraumatic distress as a primarily anxiety-based syndrome resulting exclusively from experiences of intense fear (Jordan et al., 2017; Nordstrand et al., 2019). Rather, MI is typically described as a cluster of guilt- and shame-based responses to acts that transgress deeply held moral beliefs and expectations (Litz et al., 2009). These types of experiences are often referred to as potential morally injurious events (pMIEs), and distinctions are often made between commission (perpetration), omission, and witnessing moral transgressions (Jinkerson, 2016). However, recent studies have found contradictory results regarding impact of typical commission war zone pMIEs such as killing the enemy, indicating that killing enemy combatants is not intrinsically morally injurious and may even buffer against PTSD (Cesur et al., 2013; Porter et al., 2018; Shea et al., 2017).

In this study, we aimed to develop and validate a new scale by considering the frequency and severity of both danger-based and nondanger-based events, including pMIEs (perpetration/commission, omission, and witnessing pMIEs), using initial responses from soldiers in Afghanistan and Lebanon, reflecting the experiences of soldiers operating in modern war zones. Inclusion of pMIEs is critical as a narrow focus on danger-based and fear-eliciting war zone stressors may oversimplify the complexity of major provocations in a soldier’s deeply held moral beliefs and values. Specifically, such a narrow focus in existing scales and the oversimplification of moral beliefs and values may be a potential source of confusion and misdirection in efforts aimed at both preventing negative psychological impact after war zone stressor exposure, and attempts to treat such mental health outcomes, thereby hindering significant growth in the field of military traumatic research. Given the large number of veterans developing mental health problems from serving in the recent war zones of the middle east (Fulton et al., 2015), increased knowledge on how to identify, understand, and treat mental health disorders as result of exposure to both danger- and nondanger-based war zone stressors is a pressing concern.

## This Study

This study sought to develop a reliable and valid measure of war zone exposure among military service personnel by applying multiple exploratory and confirmatory data reduction approaches. In addition to traditional EFA, a recently proposed community detection algorithm known as EGA (Golino & Epskamp, 2017) was used to understand the dimensionality underlying a pool of target items. EGA uses undirected network models to evaluate the psychometric properties of instruments and recent simulation studies indicate that EGA performs comparably to factor analytic procedures (e.g., application of the Kaiser–Gutman criteria, scree plot test, and parallel analysis) under most conditions and outperforms these approaches in large samples (Golino et al., 2020). In addition, dimensions identified by EGA provide item-level relations understood to be how items mutually influence each other in a network system, providing more granular information regarding the patterns of endorsement across item sets. Combining the results of these exploratory approaches with traditional confirmatory factor analysis (CFA) techniques provides more robust identification of latent variables or the dimensionality underlying the data. Finally, we aimed to provide strong evidence of construct validity through examination of concurrent, discriminant, and incremental validity.

## Materials and Methods

### Participants and Procedure

To achieve our study aims, this study utilized data from a cross-sectional, postdeployment examination carried out during the fall of 2020. All Norwegian military personnel deployed to Afghanistan between late 2001 and the end of 2020 were invited to participate. In total, 6,205 gave their consent to participate in the survey, resulting in a final response rate of 67.7%. As with most studies on military populations, there was a significant overrepresentation of male (*n* = 5,693; 91.7% than female: *n* = 512; 8.3%) respondents in the study population (mean age: 41.93; standard deviation [*SD*] = 9.53). The study was planned and conducted by the Institute of Military Psychiatry (IMPS), at the Joint Medical Services of the Norwegian Armed Forces (NAFs). The identified personnel received an invitation to take part in the study by a text message inviting them to complete a web-based survey. There was an incentive to participate in the form of a lottery for 30 iPads. The data collection phase lasted from September 24 to November 24, 2020, and included two reminders for those who did not respond. The study was approved by the Regional Committee for Medicine and Health Research Ethics of South-East Norway.

### Item Generation Process

The process of generating items for the new scale started as part of an epidemiological survey in 2012. Multiple relevant literature (e.g., Figley & Nash, 2011; Fontana et al., 1992; Keane et al., 1989; King et al., 2003; Litz et al., 2009; Vogt et al., 2008) were reviewed for item construction. Based on the literature review, existing scales, and experience from previous surveys of personnel from UN peace keeping missions, we considered events that covered four different types of traumatic exposure: life threat, witnessing trauma, morally injurious events, and loss. Based on David Grossmann’s (1996) work, we also included items on perpetration (killing) to evaluate the effect of such experiences. Potential items were semantically evaluated by the project group with the aim of capturing the full range of events in each category of traumatic exposure. A list of 23 items were presented to a convenience sample of veterans to validate the questionnaire against the veterans’ experiences. Based on the veterans’ feedback, the items were adjusted to improve clarity and avoid ambiguity. All items were then included in two different veteran surveys (i.e., Afghanistan 2012 study, *N* = 4,053; and the Afghanistan Prospective Resilience study, *N* = 396), intended as measures of veterans’ traumatic stress exposure, and rated on a 5-point Likert-type scale based on the frequency of exposures as 0—“not experienced”; 1—“experienced 1–2 times”; 2—“experienced 3–12 times”; 3—“experienced 13–50 times,” and 4—“experienced 50+ times.”

Through repeated iteration in 2016 by a working group as was done in 2012, the 23 questions were expanded to 39 with new items aiming to measure a range of common warzone stressors, and stressors specific to peacekeeping operations among Norwegian veterans in Lebanon, rated on a 4-point Likert-type scale ranging from 1 (no) to 4 (yes, more than 5 times), with higher scores indicating greater exposure to warzone stressors. A modified version of the original 23-item scale was later used again in a single project (Nordstrand et al., 2019) with a view of covering both danger-based and nondanger-based warzone traumatic stressors. A second pilot group of 17 veterans was nominated by the same veterans’ associations, and again, qualitative interviews were conducted to get feedback on the generated items. Based on past experiences with using the items in different veteran populations, as well as the second pilot study, the study’s working group selected 20 final items (available in the Appendix) to be included in the 2020 survey.

### Instruments

#### Warzone Stressor Exposure

The Afghanistan 2020 veteran survey included the final 20-item warzone trauma index capturing exposure to a wide range of warzone stressors commonly occurring during military deployment. Furthermore, the typology of trauma was differentiated into five categories of military trauma, namely danger-based stressors, witnessing stressors, potentially morally injurious events, traumatic loss, and perpetration of violence. The index consists of 20 items describing distinct events such as “*I was attacked by the enemy,” “I managed dead bodies or body parts,”* or *“I participated in morally transgressive actions.”* Each item was rated on a 5-point Likert scale with the response options: 0 (not experienced), 1 (experienced 1–2 times), 2 (experienced 3–12 times), 3 (experienced 13–50 times), and 4 (experienced 50+ times), giving a total sum score range of 0 to 80. Higher scores indicate a greater exposure load.

#### Satisfaction With Life

The Satisfaction with Life Scale (SWLS; Diener et al., 1985) was used to assess participant global satisfaction with life. It is a 5-item scale designed to measure global cognitive judgments of one’s life satisfaction. Participants indicate how much they agree or disagree with each of the 5 items using a 7-point scale that ranges from 1 (*strongly disagree*) to 7 (*strongly agree*). Cronbach’s alpha was α = .92.

#### DSM-5 Posttraumatic Stress

Posttraumatic symptoms related to traumatic events experienced during Afghanistan deployment were assessed with the 20-item Posttraumatic Check List-5 (PCL-5; Blevins et al., 2015). The PCL5 items are scored on a 4-point Likert-type scale from 0 (not at all) to 4 (extremely), where higher scores indicate more severe symptoms of PTSD (α = .95).

#### Posttraumatic Stress Symptoms

The 10-item Posttraumatic Symptom Scale (PTSS-10; Holen et al., 1983) was used to measure posttraumatic stress symptoms during the last 7 days. Each item has a 7-point Likert-type scale ranging from 1 (never/rarely) to 7 (very often), where higher scores indicate greater symptoms of posttraumatic stress (α = .91).

## Data Analyses

The sample was randomly divided into three, including Sample 1 (EFA; *N* = 2,013), Sample 2 (EGA; *N* = 2,075), and Sample 3 (CFA; *N* = 2,117). All analysis codes and Supplemental Material can be found in the Open Science Framework osf.io/xmsq4.

### EFA—Sample 1

To evaluate the number and factors underlying the data, we applied EFA to all 20 items. The following guidelines were employed for item and factor selection, including Kaiser–Gutman eigenvalues greater than 1, scree plot test, and parallel analysis. Prior to performing the EFA, items’ correlation matrix was inspected, and the assumptions of sampling adequacy were investigated. Inspection of the frequency distribution plots in the Supplemental Material (Fig. S1) showed that these data were characterized by an asymmetric, skewed response distribution with many observations at the floor or within the lowest region of the scale, thus greatly affecting the assumption of linearity and interval scale, a feature common to Likert-type scales (Bishop & Herron, 2015; Brown, 2015), which can seriously distort and produce inconsistent factor structures (Soto et al., 2008). Consequently, the factor analysis was performed using the weighted least square estimator (WLS) with the *fa* function in the *psych* package (Revelle, 2022), requesting polychoric correlations in RStudio. The polychoric correlation matrix is displayed in Fig. S2 in the Supplemental Material.

### EGA—Sample 2

To further evaluate the number and dimensionality underlying the data, we applied EGA using the *EGAnet* package (Golino & Christensen, 2019). The EGA estimates the number of dimensions using graphical least absolute shrinkage and selection operator based on the extended Bayesian information criteria (EBICglasso) and a weighted network community detection algorithm by performing a series of random walks to identify item clusters that are strongly connected to each other. The robustness of estimated item clusters from the EGA was evaluated through item stability and structural consistency, using 1,000 bootstrap iterations in the *bootEGA* function to estimate the median number of communities and their item composition (Christensen & Golino, 2019). Finally, we also computed network loadings, which are similar to factor loadings when the data-generating model is a common cause model and represents standardized sum of each item’s connections and contributions to a particular cluster (Christensen & Golino, 2021; Christensen et al., 2020). Effect sizes for network loadings are .15 (small), .16 to .25 (moderate), and .26 to .35 (large). Network loadings higher than .35 correspond to traditional factor loadings higher than .70 (Christensen & Golino, 2021). Based on recommendations in the factor analytic and network psychometric literature, we set three criteria for final item selection to construct the scale. Items were excluded for (a) exhibiting substantial cross-loading (λ≥ .30) in the EFA and low item stability or network loading in the EGA, (b) explained variance less than 20% (i.e., *λ*≤ .45) in the EFA and showing low item stability in the EGA, and (c) less than two items loading onto a factor in the EFA or clustering within a community in the EGA.

### CFA—Sample 3

The final solution generated by both the EFA and EGA was subjected to CFA in Sample 3, with Mplus version 8.8 (Muthén & Muthén, 1998–2023) using a Probit link function with the robust weighted least square estimator (WLSMV). To provide further evidence of construct validity, the average variance extracted (AVE) for each factor was evaluated against its correlation with the other factors (Cheung & Wang, 2017; Fornell & Larcker, 1981). Evidence of discriminant validity was confirmed when the AVE was larger than the squared correlation between factors (Fornell & Larcker, 1981). Finally, incremental validity was tested with saved factor scores corrected for measurement error to determine the additional contribution of the total score as well as subscales in a stepwise regression as separate predictors of *DSM*-5 posttraumatic stress symptoms over and above levels of posttraumatic stress during the last 7 days.

## Results

### Exploratory Factor Analyses—Sample 1

The Kaiser–Meyer–Olkin (KMO) verified the sampling adequacy for the analysis at KMO = .88, and individual KMO values except Item 4 were all above the threshold of ≥ .70 (Tabachnick & Fidell, 2014). Bartlett’s test of sphericity indicated items were acceptably correlated for factorability, χ^2^(90) = 12,462.52, *p* < .001. The first four eigenvalues were 8.72, 1.87, 1.24, and 0.95, and in combination explained 64% of the variance. The scree plot test was ambiguous as with parallel analyses (Figs. S3A and S3B in the Supplemental Material). In a final run requesting four factors (factor loadings: λ≥ .30), five items (i.e., 2, 5, 7, 11, and 13) showed cross-loadings while Item 4 loaded onto only one factor, and Item 1 did not reach .30 loading. Table 1 shows the final EFA solution with oblique rotation.

**Table 1:** Summary of Exploratory Factor Analysis Results and Exploratory Graph Analysis Results.

		Exploratory factor analysis				Exploratory graph analysis					
		Oblique rotated factor loadings						Network loadings based on EGA communities			
Items		One	Two	Three	Four	WC	Item stability, %	One	Two	Three	Four
8	I shot, directed, or led fire at the enemy	1.00				2	100	.50			
9	I have, or think I have, taken a life in the service	0.92				2	100	.34			
3	I was attacked (e.g., shelled, IED, suicide bombs, and friendly fire)	0.84				2	100	.30			
6	I was almost killed/seriously injured (“close call”)	0.81				2	99	.32			
13	I witnessed the moment when someone was seriously injured or killed	0.54			.39	2	56				
10	I had a moment where I thought I was going to die	0.52				2	78				
7	I operated in areas of high threat level (e.g., IED and hostile activity)	0.72		−.37		2	74				
2	I was in a very threatening situation (e.g., being threatened with a gun and angry people)	0.43			.34	2	75				
12	I saw seriously injured or dead people				.83	3	100			.43	
14	I saw seriously ill, injured, or dead children				.78	3	100			.35	
17	I looked after or otherwise handled dead bodies or body parts				.78	3	99			.18	
11	I felt the suffering and distress of the civilian population close to me		.34		.43	3	97			.21	
15	Someone close to me was seriously injured in the service				.37	4	100				.51
16	Someone close to me lost their life in the service				.32	4	100				.51
18	I witnessed something that was morally questionable		.79			1	75		.22		
20	I was involved in something that was morally questionable		.88			1	75		.43		
19	I failed to do something that I see in retrospect that I morally should have		.65			1	75		.27		
1	I was in a serious accident (e.g., fire, car accident, and breakdown)					1	71		.18		
4	I was captured or taken over by the enemy (e.g., carjacking)			.75		1	66	.27	.29		.16
5	I was physically injured in combat	0.36		.42		1	71		.33		

*Note.* The integers (1–4) indicate communities identified by the EGA for each item. The percentage (item stability) indicate the frequency (or proportion) of times an item clustered with the EGA-identified community more than 1,000 bootstrap iterations. WC = Walktrap community.

### Exploratory Graph Analyses—Sample 2

**Figure 1.**
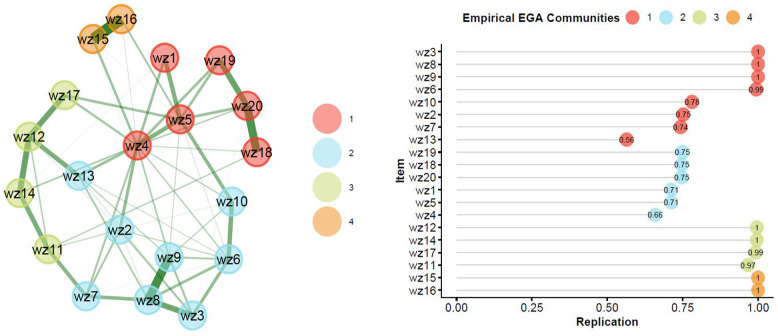
(A) Exploratory graph analysis identified communities/dimensions. (B) Average item stability plot reporting the frequency (or proportion) of times an item clustered with the EGA-identified community/dimension more than 1,000 bootstrap iterations.

The item composition of each community is reported in Table 1 and visualized in Figure 1A. Results of the structural consistency analysis revealed that a one community solution was identified 47.9% of the replications, two communities in 40.6%, three communities in 95.9%, and four communities in 100% of the bootstrap replications. Item stability analysis revealed that items mostly clustered with their EGA-identified community in the bootstrap replications. Item stability results are reported in Table 1 and visualized in Figure 1B. For example, Items 3, 8, and 9 clustered within the same EGA community 100% of all bootstrap replications. Some items, however, were not very consistently clustered with their community. For example, Items 13 and 4 clustered 56% and 66% in the bootstrap replications, and so may not be consistently uniquely identified as belonging to their respective communities. Items 15 and 16 showed very high item stability (100% each) and network loading but were only two items belonging to community four.

### Final Item Selection Based on EFA and EGA

Results from the EFA indicated that Items 4 and 5 loaded on only one factor (i.e., Factor 3) with a cross-loading Item 7. Other cross-loading items were Items 2, 5, 11 and 13. Therefore, a three-factor solution is favored based on the EFA results. Results from the EGA almost replicate the problems found in the EFA solution as Items 2, 7, 10, and 13 did not reach the network loading threshold of (λ≥ .15), and Item 4 cross-loaded in three communities. Although Item 11 cross-loaded in the EFA, it showed very high item stability in its community with very high network loading and was, therefore, included in the final selection. Items 15 and 16 had less than 20% of variance explained in the EFA and were the only two items in community four with cross-loading Item 4 from the EGA.

Within-community clustering in the EGA matched the factor solution in the EFA when low stability and problematic network loading items or cross-loading items are excluded. Thus, the pattern of item-community network loadings in the EGA was mostly consistent with the pattern of item-factor loading results in the EFA.

Based on the three criteria for final item and factor selection, results from both EFA and EGA-identified Items 3, 6, 8, 9, and 10 as belonging to one factor or community called *Personal threat factor*. Similarly, Items 11, 12, 14, and 17 belonged to one factor or community called *Traumatic witnessing*. Finally, Items 18, 19, and 20 belonged to one factor or community called MI. Overall, the results identified a *12-item, three-correlated factor structure of the Warzone Stressor Exposure Index (WarZEI)*, which can be found in Appendix A.

### CFA and Psychometric Properties of WarZEI—Sample 3

The 12-item, three-factor model returned more than adequate fit to the data, χ^2^ = 449.150, *df* = 51, *p* < .001; Standardized Root Mean Square Residual (SRMR) = .054; Root Mean Square Error of Approximation (RMSEA) = .061 (90% confidence interval [CI] = 0.056, 0.066); Comparative Fit Index (CFI) = .982; Tucker-Lewis Index (TLI) = .977. Compared with a unifactorial model testing the assumption of no construct separability, the model failed to reach acceptable fit to the data, χ^2^ = 2,237.282, *df* = 54, *p* < .001; SRMR = .127; RMSEA = .138 (90% CI = 0.133, 0.143); CFI = .902; TLI = .880. The three-factor model solution was, therefore, retained. We further systematically inspected localized areas of ill fit including the standardized pattern coefficients (λ) and explained variances (*R*^2^) to determine the substantive fit of the three-factor model. Item loadings ranged between *λ* = .72 to .96, and the explained variance ranged between *R*^2^ = 47% to 93%, which were very good. The factor structure with standardized loadings is contained in Table 2. Internal consistency estimate using Cronbach’s alpha was greater than .60 for each factor, although only slightly higher for MI (Table 2).

**Table 2:** Standardized Factor Loadings and Factor Correlations Based on the Confirmatory Factor Analysis

Personal threat		Personal threat	Traumatic witnessing	Moral injury
3 I was attacked (e.g., shelled, IED, suicide bombs, and friendly fire)	.86			
6 I was almost being killed/seriously injured (“close call”)	.88			
8 I shot, directed, or led fire at the enemy	.96			
9 I have, or think I have, taken a life in the service	.95			
10 I had a moment where I thought I was going to die	.72			
Traumatic witnessing				
11 I felt the suffering and distress of the civilian population close to me		.69		
12 I saw seriously injured or dead people		.92		
14 I saw seriously ill, injured, or dead children		.76		
17 I looked after or otherwise handled dead bodies or body parts		.78		
Moral injury				
18 I witnessed something that was morally questionable			.79	
19 I failed to do something that I see in retrospect that I morally should have			.79	
20 I was involved in something that was morally questionable			.90	
Factor correlations				
Correlations with traumatic witnessing	.59***			
Correlations with moral injury	.49***	.57**		
Composite reliability (factor rho coefficient, *ρ*)		.94	.88	.87
Cronbach’s alpha		.85	.73	.62
Average variance extracted (AVE)		.77	.63	.51

*** *p* < .001; **p < .01.

### Concurrent and Discriminant Validity

Each factor correlated significantly positively with a measure of *DSM*-5 symptoms of posttraumatic stress (personal threat, *r* = .30, *p* < .001; traumatic witnessing, *r* = .27, *p* < .001; and MI, *r* = .33, *p* < .001), thus providing evidence to support acceptable concurrent validity, although correlations were not strong. Discriminant validity was supported for all the factors as the AVE values compared with the squared correlations between factors indicated no issues. Additional results also showed that personal threat (β = .08, *p* < .001), traumatic witnessing (β = .09, *p* < .05), and MI (β = .18, *p* < .001) uniquely predicted posttraumatic stress symptoms. In addition, although the factors were significantly negatively correlated with life satisfaction, the correlations were noticeably smaller and near zero (personal threat, *r* = −.08, *p* < .01; traumatic witnessing, *r* = −.08, *p* < .01; and MI, *r* = −.16, *p* < .01), thus providing acceptable evidence of discriminant validity. Factor correlations are presented in the bottom of Table 2 with each factor’s AVE values.

### Incremental Validity

We examined the predictive accuracy of WarZEI in accounting for additional variance in *DSM*-5 symptoms of posttraumatic stress scores above and beyond posttraumatic stress symptoms during the last 7 days. Personal threat predicted *DSM*-5 symptoms of posttraumatic stress (standardized: β = .18, *SE* = .06, *t* = 12.49, *p* < .001), accounting for additional variance (Δ*R*^2^ = .03) above posttraumatic stress symptoms during the last 7 days (β = .73, *SE* = .02, *t* = 50.35, *p* < .001; *R*^2^ = .58). MI also predicted *DSM*-5 symptoms of posttraumatic stress (β = .14, *SE* = .13, *t* = 9.11, *p* < .001; Δ*R*^2^ = .02) above posttraumatic stress symptoms during the last 7 days (β = .73, *SE* = .02, *t* = 48.58, *p* < .001; *R*^2^ = .58). Similarly, traumatic witnessing predicted *DSM*-5 symptoms of posttraumatic stress (β = .13, *SE* = .07, *t* = 8.44, *p* < .001; Δ*R*^2^ = .02) and above posttraumatic stress symptoms during the last 7 days (β = .74, *SE* = .02, *t* = 49.98, *p* < .001; *R*^2^ = .58). Thus, support for incremental validity was found for each factor, although additional variances explained were not much.

## Discussion

The most widely used scales assessing military personnel’s exposure to traumatic stressors in war zone mainly focus on fear, horror, and danger-based threats of death and bodily harm while engaged in combat actions, with comparatively few items indexing exposure to nondanger-based warzone stressors. In response, this study has developed and validated a new index to assess exposure to warzone stressors that captures a wide range of exposure to both danger-based and nondanger-based stressors. Developed from previous iteration of 20 items, the 12-item index is tentatively referred to as WarZEI, covering broad domains of personal threat, perpetration, witnessing, personal loss, and moral challenges. Its name aims to contrast the typical label of a combat stressor index, and the rationale for this being the emerging evidence showing how a range of warzone experiences can precipitate negative post-trauma mental health trajectories. Moreover, a second tier has been added to the questionnaire, which was not part of the original scale, asking respondents to rate how severely each stressor category impacted them since individual differences in responding to traumatic stressors mean that for some people only a single exposure could constitute greater severity while for some others it could take more than one or repeated exposures to reach greater severity.

Initial EFA results appeared to support a four-factor model, but with some problematic item loadings. Upon further inspection, the pattern of item-factor loadings in the EFA was found to align very well with the pattern of item-community network clustering in the EGA for retaining a 12-item, three-factor model. Results of subsequent CFA indicated that the three-factor model was well supported with model fit indices reaching thresholds of excellent model-to-data consistency. Furthermore, the factors were significantly positively correlated and related to warzone stressors, namely *personal threat* (α = .94), *traumatic witnessing* (α = .80), and MI (α = .68). As contained in Table 2, moderate factor correlations show that the factors are not too highly overlapping although related and thus differ in important ways, providing support for heterogeneity and multidimensional nature of war zone stressors. The newly developed index is contained in Appendix A. Taken as a whole, the items, when combined with anchors to measure frequency and severity, differ from existing scales and insofar as they (a) include items to measure exposure to danger-based stressors, nondanger-based stressors, and potentially morally injurious events comprising both perpetration and witnessing; (b) were developed using an item pool based on several initial responses of soldiers deployed to combat action in Afghanistan and Lebanon; and (c) were selected and evaluated based on theoretical framing of traumatic warzone stressors (e.g., Figley & Nash, 2011; Fontana et al., 1992; Litz et al., 2009; Nordstrand et al., 2019, 2020; Vogt et al., 2008), bearing in mind the heterogeneity in war zone stressors and their differential impact on subsequent psychological sequelae. Our inspection of the data from the original measure revealed that participants infrequently reported experiencing 13–50 events, and instances of experiencing 50 or more events were even rarer. The majority of responses fell into the lower categories of the original scale, specifically *“not experienced,” “experienced 1–2 times,”* or *“experienced 3–12 times.”* These categories represent the lower end of the scale, suggesting that the original response options were not capturing meaningful variability in participant experiences. Given this distribution, the decision was made to refine the response options in the newly developed short version. The revised version more accurately reflects the observed data distribution by collapsing the higher frequency categories, which were rarely endorsed, and providing finer distinctions within the lower range where most responses occurred. The updated response options are as follows: *0 (never experienced), 1 (experienced 1 time), 2 (experienced 2–4 times), 3 (experienced 5–9 times), and 4 (experienced 10 or more times)* with the severity tier also scored as *0 (not at all severe), 1(a little severe), 2(somewhat severe), 3(severe), and 4(very severe).* This adjustment enhances the WarZEI’s sensitivity in capturing the frequency and severity of event experiences, particularly within the range where most participants’ responses fall. By concentrating on the lower frequency categories, the new scale better differentiates between varying levels of exposure, leading to more precise data and improving the overall reliability and validity of WarZEI.

The most unique feature of the newly developed index is that it assesses the *frequency* and *severity* of traumatic stressors since individual differences in responding to traumatic stressors mean that for some people only a single exposure could constitute greater severity while for some others it could take more than one or repeated exposures to reach greater severity. Furthermore, for some people, initial exposure to traumatic stressors may operate as inoculation if they successfully overcome it, while this may be untrue for some others (Garrison, 1991). Benefits of the newly developed index include the fact that fewer items were retained based on multiple exploratory data reduction techniques and confirmatory approaches, which means this index is more parsimonious in time administration and more precise in construct definition as longer scales are vulnerable to inclusion of ambiguous items (Allen et al., 2022). Test-takers in combat exposures or after combat action may have depleted cognitive and emotional resources, which means shorter scales are more suitable for administration to lessen the burden of survey responses. This also means that response rate could be higher and the preparation of the survey whether online or through paper-and-pen will require less data preparation cost as with shorter scales (Allen et al., 2022).

### Personal Threat

The personal threat factor assesses threats to personal life comprising fear-based events that could be dangerous and horrific (e.g., near-death events in combat action). In this study, personal threat predicted *DSM*-5 posttraumatic stress symptoms when controlling for posttraumatic stress symptoms during the last 7 days while it also showed significant negative correlations with satisfaction with life. Personal threat has been found to be significant predictor of posttraumatic stress symptoms in a previous sample of Norwegian military personnel deployed to Afghanistan between 2001 and the end of 2011 (Nordstrand et al., 2019). In another study, exposure to personal life threat predicted PTSD symptoms of hyperarousal (Shea et al., 2017). However, different experiences of personal life threats such as incoming fire did not predict hyperarousal symptoms, but exposure to mines or booby traps did in a study of the relationship between combat experiences and combat-related PTSD (Pietrzak et al., 2011). Contrary, the threat to personal life has also been found not to predict PTSD symptom of hyperarousal although it predicted current feeling of fear (Stein et al., 2012).

### Traumatic Witnessing

As previously noted, peritraumatic fear may not always be present, and the threat to personal life or body may not be the most stressful part in a war zone, thus raising the need to account for nondanger-based stressors. Traumatic witnessing assesses sensory impressions of death or major suffering of others by seeing, touching, or smelling as well as learning about the death or injury of someone close (e.g., witnessing the aftermath of terrorist attack on a civilian target or seeing and handling of unit members’ dead bodies) (Fontana et al., 1992; Pietrzak et al., 2011; Stein et al., 2012). In this study, traumatic witnessing predicted *DSM*-5 posttraumatic stress symptoms over and above posttraumatic stress symptoms during the last 7 days. The current results are consistent with previous studies which found that traumatic witnessing positively predicted posttraumatic stress symptoms, anxiety, and depression symptoms as well as insomnia (Nordstrand et al., 2019). Furthermore, other studies have found traumatic witnessing to be associated with depression (Shea et al., 2017), and that personally witnessing someone from one’s unit being seriously wounded or killed had a stronger association with PTSD than experiences involving personal life threat (Pietrzak et al., 2011). As a side note, previous studies have shown that nondanger-based stressors also tend to precipitate a posttraumatic deprecation along nonclinical psychological domains such as existential beliefs and values, interpersonal involvement, and self-confidence (Nordstrand et al., 2019). In contrast, danger-based stressors are more likely to be associated with positive personal changes in the same domains, often referred to as posttraumatic growth (Maguen et al., 2006). Such findings are congruent with the current results and indicate that the disparate psychological impact of the stressor categories in the WarZEI likely extends beyond clinical mental health symptoms.

### Moral Injury

Major violations of an individual’s values and morality without threat to personal life can also be stressful in a war zone, which the MI factor captures. This factor assesses the commission (perpetration) or omission, or the witnessing of events that invoke moral transgressions (Litz et al., 2009), stemming from the provocations in a soldier’s deeply held moral beliefs and values (e.g., involvement in collateral damage that results in civilian casualties). pMIEs were found to predict *DSM*-5 posttraumatic stress symptoms over and above posttraumatic stress symptoms during the last 7 days. This accords with results from previous studies which found that moral challenges (relevant to our definition of MI) positively predicted posttraumatic stress symptoms, anxiety, and depression symptoms as well as insomnia (Nordstrand et al., 2019). Like traumatic witnessing, MI also invokes guilt and shame that have been linked with increased debilitating psychological distress and depressive symptoms (Stein et al., 2012), although other researchers have noted that the probability of developing PTSD following these events is lower relative to other traumas (Kessler et al., 2017; Wisco et al., 2014).

### Limitations and Recommendations for Future Work

All services in the NAF mission in Afghanistan were voluntary; moreover, there were stringent requirements on previous service length (18 months minimum). Applicants for service in Afghanistan also had to pass several screenings for physical and psychological health, as well as proficiency tests with regard to military skills. There is a wide variability between different country military organizations in terms of selection, training, health support, and missions (e.g., Gjerstad et al., 2020; Hougsnæs et al., 2017; Morgan et al., 2019; Stevelink et al., 2018; Zamorski et al., 2014). Thus, it is important to be mindful of the possible limitations to the generalizability of the current results due to idiosyncrasies in the NAF selection and training of soldiers for combat missions. Despite the benefits of a shorter scale, item reduction could also lead to limited breadth and scope of the constructs intended for assessment and thus introduce validity concerns. We attempted to mitigate this by selecting items based on several years of theoretical work and initial responses of soldiers deployed to combat action in Lebanon and Afghanistan and using multiple data reduction approaches. It should be noted that the items used to capture pMIEs in the WarZEI do not cover all elements sometimes identified as potentially morally injurious. As an example, experiences of systemic betrayal are often regarded as pMIEs; however, due to our ambition to create a short-form index, we choose to include only items covering what the MI literature identifies as the core features of pMIEs (e.g., Hall et al., 2022). It was observed that the distribution of responses in the data was skewed. When floor effects are high, it can distort and produce inconsistent factor structures (Soto et al., 2008) due to violations of linearity and interval scale, a feature common to Likert-type scales (Bishop & Herron, 2015; Brown, 2015). As in this study, future studies should correctly account for ordinality of scale items and the violations of linearity by employing appropriate categorical estimation methods (e.g., weighted-least squares, WLS) or compare results from both categorical and continuous (e.g., Maximum likelihood, ML) estimation methods and to report results from the categorical estimation method when differences appear in the two estimation methods (Anyan et al., 2021; Finney et al., 2016; Sass et al., 2014). The consequences for ignoring the ordinality of scale items can be profound, including incorrect parameter estimates and test statistics, factors that are artifacts of item extremeness, inconsistent factor structures, and attenuated relationship between factors (Brown, 2015).

## Conclusion

The development and validation of the WarZEI resulted in a 12-item, three-correlated factor model (*personal threat*, *traumatic witnessing*, and MI) for assessing exposure to war zone stressors that allows for heterogeneity and the multidimensional nature of exposure to warzone stressors. Inherent in previous scales are potential source of confusion and misdirection in the efforts to prevent negative psychological impact after war zone stressor exposure and attempts to treat such mental health outcomes due to the narrow focus of the scales. The WarZEI attempts to minimize or prevent these critical concerns by broadening the scope of war zone stressors while making judicious use of item content to reach a shorter and more precise scale that can grow the field of military traumatic research. More importantly, the WarZEI aims to demonstrate that the items cover the theoretical framing and can reliably and validly assess exposure to war zone stressors.

## Supplemental Material

sj-docx-1-asm-10.1177_10731911241298083 – Supplemental material for Development and Validation of a Brief Warzone Stressor Exposure Index Supplemental material, sj-docx-1-asm-10.1177_10731911241298083 for Development and Validation of a Brief Warzone Stressor Exposure Index by Frederick Anyan, Andreas Espetvedt Nordstrand, Odin Hjemdal, Line Rønning, Ann Hergatt Huffman, Laura K. Noll, Christer Lunde Gjerstad, Robert E. Wickham and Hans Jakob Bøe in Assessment

## Appendix A

**Table:**

Warzone Stressor Exposure Index (WarZEI)											
*Below are some events which you may have experienced or witnessed. Indicate how many times you experienced or witnessed these events and how severe they were.*											
		*How often did you experience, or witness events described on the left*					*Overall, how severe was the event you experience or witness?*				
		Never	1 time	2–4 times	5–9 times	10 or more times	Not at all severe	A little severe	Somewhat severe	Severe	Very severe
1	I was attacked (e.g., shelled, IED, suicide bombs, and friendly fire)										
2	I felt the suffering and distress of the civilian population close to me										
3	I witnessed something that was morally questionable										
4	I was almost killed/seriously injured (“close call”)										
5	I saw seriously injured or dead people										
6	I failed to do something that I see in retrospect that I morally should have										
7	I shot, directed, or led fire at the enemy										
8	I saw seriously ill, injured, or dead children										
9	I was involved in something that was morally questionable										
10	I have, or think I have taken a life in the service										
11	I looked after or otherwise handled dead bodies or body parts										
12	I had a moment where I thought I was going to die										

## Appendix B

**Table:** All 20 Items Included in the 2020 Survey

1	I was in a serious accident (e.g., fire, car accident, and breakdown)
2	I was in a very threatening situation (e.g., being threatened with a gun)
3	I was attacked (e.g., shelled, IED, suicide bombs, and friendly fire)
4	I was captured or taken over by the enemy (e.g., carjacking)
5	I was physically injured in combat
6	I was almost being killed/seriously injured (close call)
7	I operated in areas of high threat level (e.g., IED and hostile activity)
8	I shot, directed, or led fire at the enemy
9	I have, or think I have, taken a life in the service
10	I had a moment where I thought I was going to die
11	I felt the suffering and distress of the civilian population close to me
12	I saw seriously injured or dead people
13	I witnessed the moment when someone was seriously injured or killed
14	I saw seriously ill, injured, or dead children
15	Someone close to me was seriously injured in the service
16	Someone close to me lost their life in the service (Loss)
17	I looked after or otherwise handled dead bodies or body parts
18	I witnessed something that was morally questionable
19	I failed to do something that I see in retrospect that I morally should have done
20	I was involved in something that was morally questionable
