# Supplementary material for: Development and Validation of a Brief Warzone Stressor Exposure Index
Source: Assessment. 2024 Dec 5;32(8):1235–49. doi: 10.1177/10731911241298083 (PMC12579719; doi:10.1177/10731911241298083)
Supplement: sj-docx-1-asm-10.1177_10731911241298083 – Supplemental material for Development and Validation of a Brief Warzone Stressor Exposure Index [file sj-docx-1-asm-10.1177_10731911241298083.docx]

**SUPPLEMENTARY MATEIRAL**

**Title:** Development and Validation of a brief War Zone Stressor Exposure Index (WarZEI)

**Journal:** Assessment

**Data preparation and screening**

Sample for EFA was found to be non-normally distributed (Mardia’s multivariate skewness, *M_S_* = 722732.98, *p* < .05; Mardia’s multivariate kurtosis, *M_K_* = 1685.58, *p* < .05) as was the case for the CFA sample (*M_S_* = 24980.61, *p* < .05; *M_K_* = 180.01, *p* < .05). Weighted least square estimation is robust against non-normality in the data. The sample for EGA was found to be non-normally distributed (*M_S_* = 1685.58, *p* < .05; *M_K_* = 234.67, *p* < .05), therefore cases that were considered substantial multivariate outliers were removed (*n* = 163) based on Mahalanobis distance because they would disproportionately influence results.


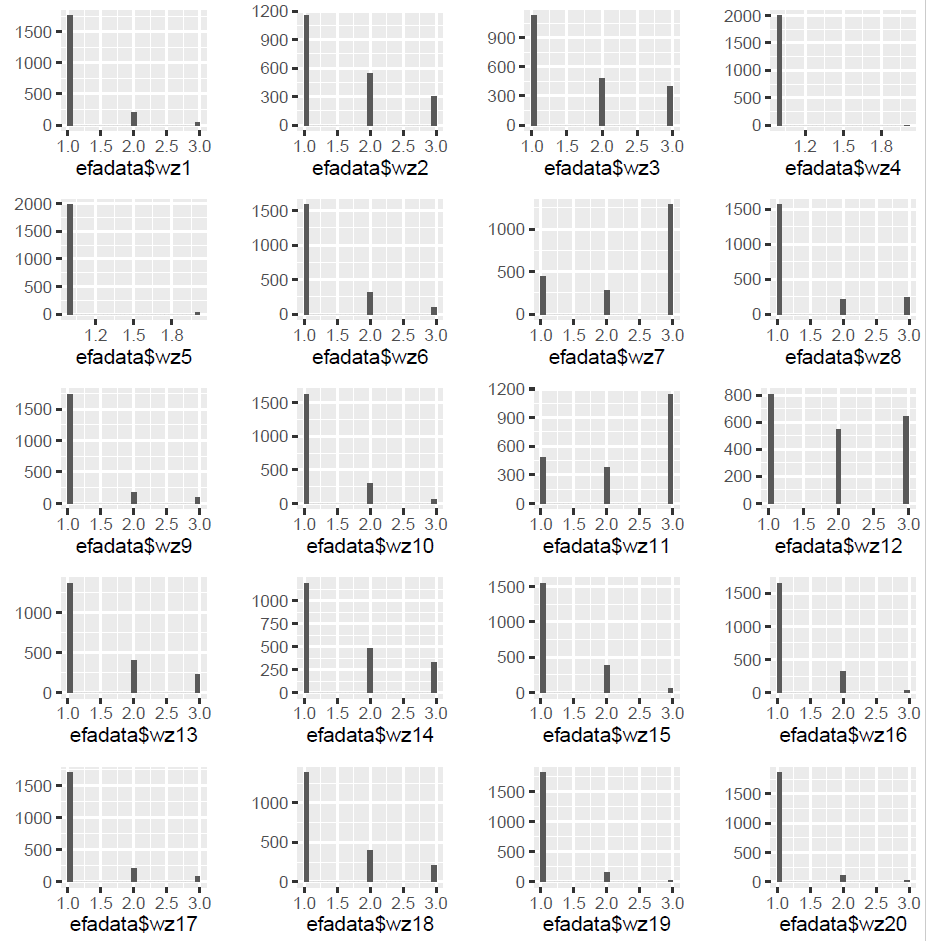


**Fig. S1:** Frequency distribution plots for all 20 items


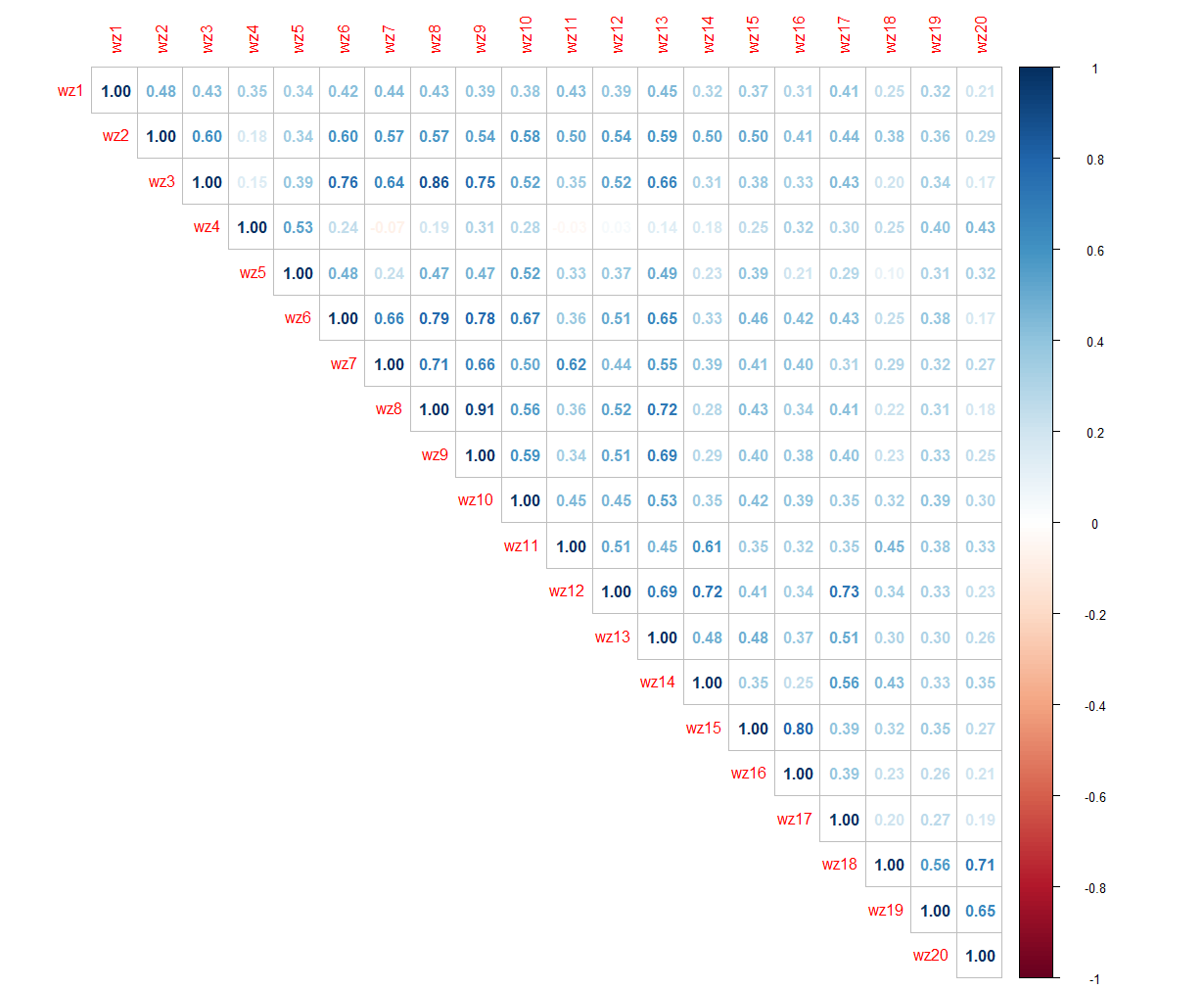


**Fig. S2A:** 20-item polychoric correlation matrix.


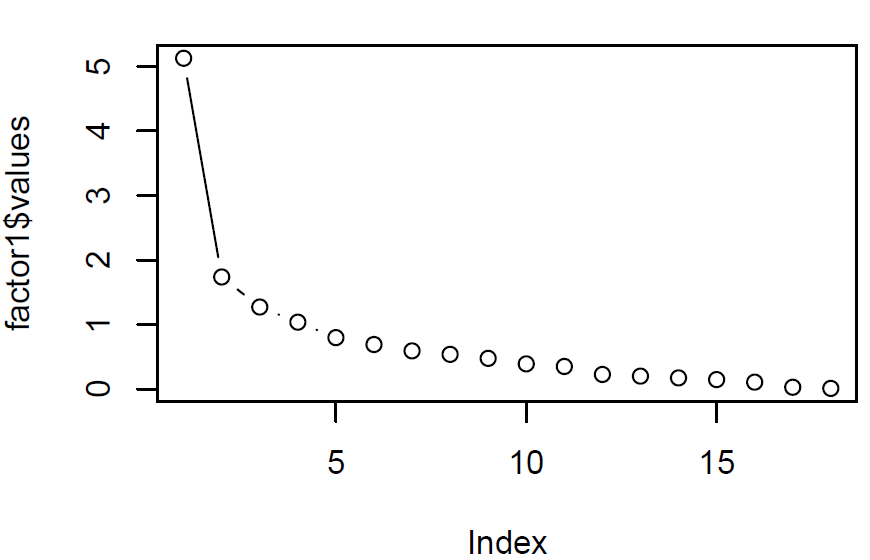


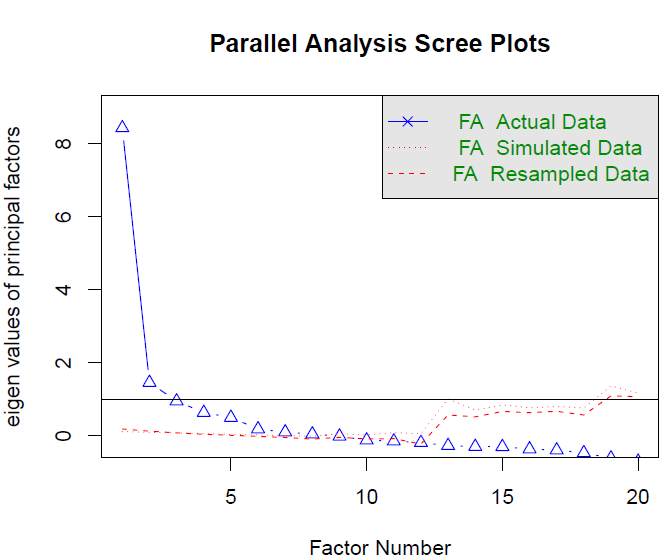


**Fig. S3A:** Scree plot. **Fig. S3B:** Parallel Analysis plot

| **Table S1: Supplemental to Convergent and discriminant validity tests based on correlations** | | | | | |
| --- | --- | --- | --- | --- | --- |
|  | Personal threat | Traumatic witnessing | Moral injury | Posttraumatic stress disorder | Satisfaction with life |
| Personal threat | -- |  |  |  |  |
| Traumatic witnessing | .71^**^ | -- |  |  |  |
| Moral injury | .64^**^ | .72^**^ | -- |  |  |
| Posttraumatic stress disorder | .30^**^ | .27^**^ | .33^**^ | -- |  |
| Satisfaction with life | -.11^**^ | -.09^**^ | -.16^**^ | -.50^**^ | -- |
| **Correlation is significant at the 0.01 level (2-tailed). | | | | | |
